# Effects of Serum Metabolites on the Pancreatic Transcriptome in Acute Acalculous Cholecystitis

**DOI:** 10.1155/2021/2368571

**Published:** 2021-12-08

**Authors:** Yuanyuan Sun, Qiang Wang, Chenjun Hao, Dongbo Xue

**Affiliations:** Department of General Surgery, Laboratory of Hepatosplenic Surgery, Ministry of Education, The First Affiliated Hospital of Harbin Medical University, Harbin 150001, China

## Abstract

**Background:**

To provide a basis for the diagnosis and treatment of acalculous biliary pancreatitis, this study investigated the impact of serum metabolites on the pancreatic transcriptome in acute acalculous cholecystitis (AAC).

**Methods:**

Fourteen rabbits were randomly divided into two groups (a normal control group of 7 rabbits and an AAC group of 7 rabbits), blood was collected from the 14 rabbits, and metabolomic analysis was performed through ^1^H NMR. Two pancreatic tissue chips of the AAC group and the normal control group were prepared and sequenced. We utilized the limma package of R software, the DAVID database, the STRING database, Cytoscape software, and the CFinder analysis tool to perform differential expression gene analysis, gene function enrichment analysis, protein interaction network (PPI) construction, and network module mining, and we performed gene enrichment analysis in each module.

**Results:**

Serum metabolism analysis showed that in AAC, the metabolism of sugar, lipids, and protein, that is, the three major nutrients, was affected to varying degrees, and levels of serum trimethylamine N-oxide (TMAO) increased. Bioinformatic methods were utilized to identify a total of 183 differentially expressed genes and 3 key genes. Enrichment analysis showed that differentially expressed genes were significantly enriched in cation transport, the inflammatory response, the NF-*κ*B pathway, and the cancer signaling pathway.

**Conclusion:**

Metabolomic analysis and functional analysis of 3 key genes demonstrated that abnormal serum metabolites affected the pancreatic transcriptome and induced a sensitive state of inflammation in the pancreas. These metabolites may represent important targets for future research on the pathogenesis, clinical diagnosis, and treatment of noncalculous biliary pancreatitis.

## 1. Introduction

Acute pancreatitis (AP) is a conditions marked by acute pain in the abdomen; this disease not only affects the pancreas but also invades multiple organs. AP exhibits high morbidity and mortality. AP is primarily caused by cholelithiasis, excessive alcohol intake, hyperlipidemia, endoscopic retrograde cholangiopancreatography, and various drugs. These factors cause dysfunctional cell pathways and organelle pathology followed by the activation of pancreatic enzymes in the pancreas, which further leads to systemic inflammatory response syndrome [1, 2]. Biliary pancreatitis is primarily caused by stones incarcerated at the end of the common bile duct, which causes the pressure in the bile duct and pancreatic duct to increase, and the bacteria and inflammatory mediators in the bile flow back into the pancreatic duct, causing the activation of pancreatin in the pancreatic duct and the occurrence of acute pancreatitis. Acute pancreatitis caused by acalculous biliary factors is observed most commonly in biliary tract infections, congenital biliary tract diseases, or pathological changes of the Oddi sphincter [3], and its specific pathophysiological mechanism has not been elucidated. Acute pancreatitis caused by acalculous biliary factors is not significantly different from pancreatitis caused by other causes in terms of the grade, outcome, and complications of acute pancreatitis, but it has particularities regarding its pathogenesis and management principles. Studying the pathophysiological mechanism of acute pancreatitis caused by acalculous biliary factors may facilitate the precise diagnosis and treatment of the disease. Among other acalculous causes of acute pancreatitis, the pathophysiological mechanism of this disease is mostly accompanied by pathological changes in the pancreas caused by abnormal metabolites. For example, alcohol metabolites can cause acute acinar cell damage and subsequent cytokine release, which ultimately leads to systemic inflammatory response syndrome [4], providing a direction for research on the pathophysiological mechanism underlying acute pancreatitis caused by acalculous biliary factors. Therefore, we investigated the effect of serum metabolites on the pancreatic transcriptome during AAC using metabolic analysis, chip sequencing, and bioinformatics, and our results provide a theoretical basis for further research studying the pathogenesis, clinical diagnosis, and treatment of acalculous biliary pancreatitis.

## 2. Materials and Methods

To study the serum metabolites in rabbits, we followed the methods of Li et al. [5]. Female rabbits weighing 1800–2200 g were purchased from the Animal Center of Harbin Medical University. The rabbits were housed in cages under a controlled temperature of 26°C and 12 h light–dark cycles, fed standard laboratory chow with water provided *ad libitum*, and given at least a week to acclimate. The rabbits were fasted overnight with free access to water before experiments. The study was designed in accordance with the guidelines for the care and use of laboratory animals in research and was approved by the Ethics and Research Committee of Harbin Medical University.

Fourteen healthy rabbits were randomly divided into two groups. The rabbits in the AAC group were anesthetized with 3% pentobarbital sodium, administered at a dose of 30 mg/kg by ear vein injection. A vertical incision of 4-5 cm was made in the middle of the abdomen to expose the gallbladder, and 0.3 ml of *Escherichia coli* was injected with a 1 ml syringe (EPEC: O15/O26/O111/O128: K67 B12/E2348, concentration: 1.0 × 10^5^/ml, obtained from the Department of Microbiology, Harbin Medical University). The rabbits in the control group were injected with an identical volume of normal saline into the gallbladder after laparotomy.

### 2.1. Serum Metabolomic Test

Three-milliliter blood samples were collected from the rabbits in both groups 72 h after the operation, and the samples were centrifuged at 1500 rpm for 5 min. The supernatants were collected and stored in a -20°C refrigerator for metabolomic analysis. The frozen serum sample was thawed at room temperature, and 400 *μ*l serum superstratum was transferred into a 5 mm nuclear magnetic tube. Next, 100 *μ*l D2O (-for lock) was added, and the solution was vibrated and fully mixed. ^1^H NMR experiments were performed on a Varian 600 spectrometer with a 599.925 Hz ^1^H resonance frequency. One-dimensional spectra were recorded by suppressing the strong water signal using a standard NOESY1DPR pulse sequence (RD-90°-t1-90°-tm-90°-ACQ). The relaxation delay (RD) was 2.5 s, the time for mixing (tm) was 100 ms, and the cumulative frequency was 64. Saturated irradiation was performed on the water at the RD and tm. A Carr-Purcell-Meiboom-Gill (CPMG) sequence was used for the transverse (T2) relaxation weighing experiment, for which the RD was 4 s, the total echo time was 100 ms, and the cumulative frequency was 256.

### 2.2. Principal Component Analysis (PCA)

The range of the spectrum interval selected by principal component analysis was the signal with a chemical shift within *δ* 0-10. The method was as follows. First, the full spectrum of each sample (*δ* 0-10) was divided into 1000 sections with 0.01 ppm intervals, the integral value of the water signal (*δ* 4.71-5.20) was removed, the remaining 950 section integral values were added and normalized, and SIMCA-13.0 software was used to perform principal component analysis (PCA) in center-scaling mode.

### 2.3. Differential Peak Screening

According to the results of the PCA between the groups, the signal peaks of the above metabolites were selected, the *T*-test between the AAC group and the control group was employed, and the difference peak was selected with a *P* value < 0.05.

### 2.4. Chip Sequencing

After the pancreatic tissue was isolated, it was placed in a cryotube, marked, quickly frozen, and stored in liquid nitrogen. For RNA extraction, the pancreatic tissue samples were removed from the liquid nitrogen tank, placed in a precooled mortar, and ground for 8-10 min; next, 2-3 ml TRIzol reagent (Invitrogen, Gaithersburg, MD, USA) was added to each mortar and grinding continued until the temperature of the mortar returned to room temperature; after that step, TRIzol reagent was transferred to a glass homogenizer, and samples were homogenized for 3-5 min and subsequently transferred to a 1.5 ml centrifuge tube, and total RNA was extracted according to the standard procedure for TRIzol reagent. Total RNA was purified by column with a NucleoSpin® RNA clean-up kit (MACHEREY-NAGEL, Germany) and was finally quantified by a spectrophotometer and subjected to quality inspection through formaldehyde denaturing gel electrophoresis.

We started with total RNA and the T7 Oligo (dT) Promoter Primer Kit containing the T7 promoter sequence as primer, and we used the CbcScript enzyme to synthesize first-strand cDNA. RNase H was used to cut the RNA in the hybrid strand into short fragments. DNA polymerase uses short RNA fragments as primers to synthesize second-strand cDNA and purify double-stranded cDNA. Using cDNA as a template, T7 Enzyme Mix was used to synthesize cRNA, and an RNA clean-up kit (MN) was used for purification. Five micrograms of cRNA, CbcScript II enzyme, and random primer were used for reverse transcription, and a PCR NucleoSpin Extract II Kit (MN) was used to purify the reverse transcription product. Taking the above reverse transcription product, we used Random Primers as the primer for KLENOW enzyme labeling, and we used the PCR NucleoSpin Extract II Kit (MN) to purify the labeled product and then drain it after purification (Cy5-dCTP or Cy3-dCTP) (GE Healthcare Cat. No. PA 55021/PA53021). The labeled DNA was dissolved in hybridization solution (2X GEx Hyb Buffer (HI-RPM), 25% formamide) and hybridized at 45°C overnight. After hybridization, the cells were first washed with 0.2% SDS and 2x SSC at 42°C for 5 min and subsequently washed in 0.2x SSC at room temperature for 5 min. After the slides were dried, an Agilent rabbit gene expression microarray was used to scan the slides and obtain the hybridization image. Feature extraction image analysis software was used to analyze the chip image and convert the image signal into a digital signal.

### 2.5. Data Processing

The limma package of R software was used to read and preprocess the data of 4 sample chips under the Agilent platform. The preprocessing process included background correction and data normalization. We downloaded the corresponding platform annotation file from NCBI GEO [6], and the annotation file number was GPL13288. We converted the probe ID to the corresponding gene ID. Afterwards, we downloaded the latest version of the gene annotation file gene2accession from the NCBI Gene database and further processed it to obtain the gene ID and gene symbol annotation files of *Oryctolagus cuniculus* species. We converted the gene ID in the expression matrix to a gene symbol. Finally, 898 gene expression values were obtained.

### 2.6. Differentially Expressed Gene Screening and Functional Analysis

Next, we continued to use the limma package to analyze the differentially expressed genes in the AAC group and the control group. In the analysis process, the corrected *T*-test provided by limma was used to calculate the significance *P* value of the differentially expressed genes. For each significantly differentially expressed gene, the differential expression *P* value < 0.05 and the fold change (FC) difference between the sample groups were not less than 1.5-fold (the absolute value of log_2_FC was not less than 0.58). We downloaded the pancreatic gene expression dataset GSE119844 of mouse acute pancreatitis, and the pancreatic gene expression dataset GSE143754 of human chronic pancreatitis, from the NCBI GEO database (https://www.ncbi.nlm.nih.gov/geo/), and then, we used limma package of R software (version 4.03) to analyze the differential expression of genes in the two datasets, respectively.

For the differential expression genes in the rabbit pancreas, we used the commonly used enrichment analysis tool DAVID [7] to analyze the Gene Ontology (GO) [8] function and KEGG [9] pathway involved in upregulated and downregulated genes. The number of parameter-enriched genes was ≥2, and the significance threshold of the hypergeometric test was a *P* value < 0.05.

### 2.7. PPI Network of Differentially Expressed Genes

The STRING [10] database was used to predict and analyze the interaction relationship between the proteins encoded by differentially expressed genes. The input gene set was differentially expressed genes, and the species was *Oryctolagus cuniculus*. The parameter PPI score was set to 0.4 (indicating medium confidence), and the protein nodes that required interaction were all differentially expressed genes. Cytoscape [11] was used to construct a PPI network.

### 2.8. Analysis of Topological Properties of PPI Network Nodes and Network Module Analysis

We combined four calculation methods of network topology properties to analyze the importance of nodes in the network. The four methods, that is, degree centrality, betweenness centrality, subgraph centrality, and closeness centrality, were employed to analyze the scores of nodes in the network. The tool used to calculate the four centrality scores of the network was the Cytoscape plugin CytoNCA [12] (parameter setting: network is without weight). In the output of CytoNCA, the higher the node score was, the more important the position in the network was, and the more likely it was to be a key node.

Based on the analysis tool CFinder [13], subnetwork mining was performed on the constructed differential gene PPI network. CFinder uses the clique percolation method (CPM) for subnetwork mining. It was necessary to set the faction value *k*, and the larger the *k* value was, the smaller the module obtained was, but the more compact the structure would be. In this instance, the *k* value in the CFinder subnet mining process was set to 4 by default. After we obtained the subnetwork modules, we used DAVID to analyze the biological functions (GO function and KEGG pathway) of each network module and screened out significantly enriched results.

## 3. Results

### 3.1. Principal Component Analysis Results for Serum Metabolites

**Figure 1:**
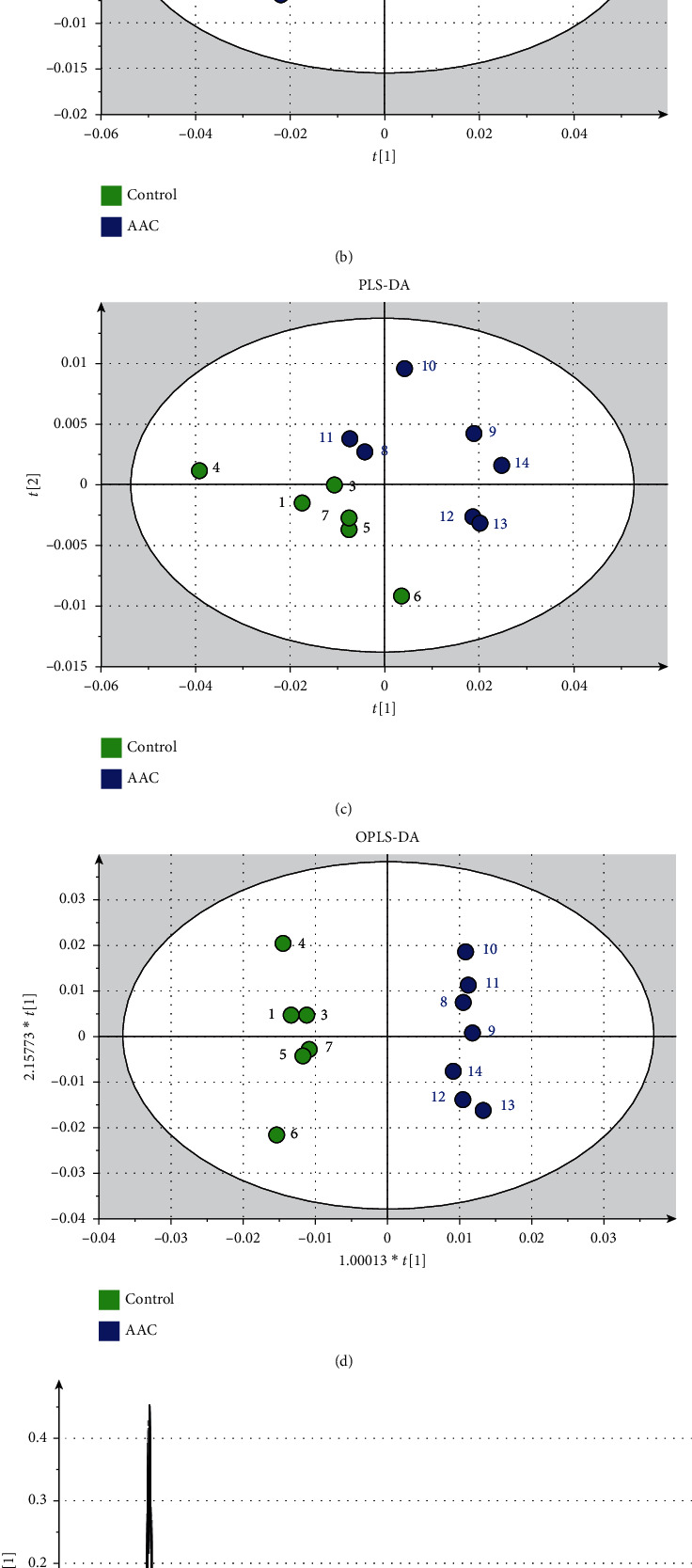
Results of ^1^H NMR analysis of rabbit serum. (a) PCA score graph results of 14 samples; (b) PCA score graph results of 13 samples (excluding sample 2); (c) 13 samples (excluding no. 2 sample) PLS-DA score chart results; (d) 13 samples (excluding no. 2 sample) OPLS-DA score chart results; (e) 13 samples (excluding no. 2 sample) PCA load chart; (f) comparison of the peak value of TMAO spectrum between the AAC group and the control group.

The PCA results of the serum metabolites of 14 samples are shown in the score chart (Figure 1(a)). From the PCA results, it could be seen that the two groups of sera could not be effectively separated, and sample no. 2 deviated considerably. In the subsequent analysis, two methods could be used to eliminate information or perform orthogonal signal correction (OSC) to further process the above results. Orthogonal correction of partial least squares-discriminant analysis (OPLS-DA) was used to introduce a categorical group variable (*Y* variable) into PCA, orthogonalize the previous variable matrix (*X* variable), and eliminate the irrelevant discriminant analysis results, as performed by *X* orthogonal and retained related variables. From PCA (Figure 1(b)), PLS-DA (Figure 1(c)), and OPLS-DA (Figure 1(d)), it could be observed that the two groups of serum could be effectively separated after removing sample no. 2.

From the load diagram (Figure 1(e)), it could be observed that the difference between the spectral signal peaks of the AAC group and the control group was mainly concentrated in such areas as fat (*δ* 0.89, *δ* 1.29, *δ* 1.57, *δ* 2.24, *δ* 5.32), glucose (*δ* 3.27, *δ* 3.42, *δ* 3.48, *δ* 3.72, *δ* 3.55, *δ* 3.84, *δ* 3.90, *δ* 4.65, *δ* 5.24), lactic acid (*δ* 1.34, *δ* 4.12), glycoprotein (*δ* 2.04), and trimethylamine N-oxide (TMAO) (*δ* 3.31).

### 3.2. Differential Peak Screening

**Figure 2:**
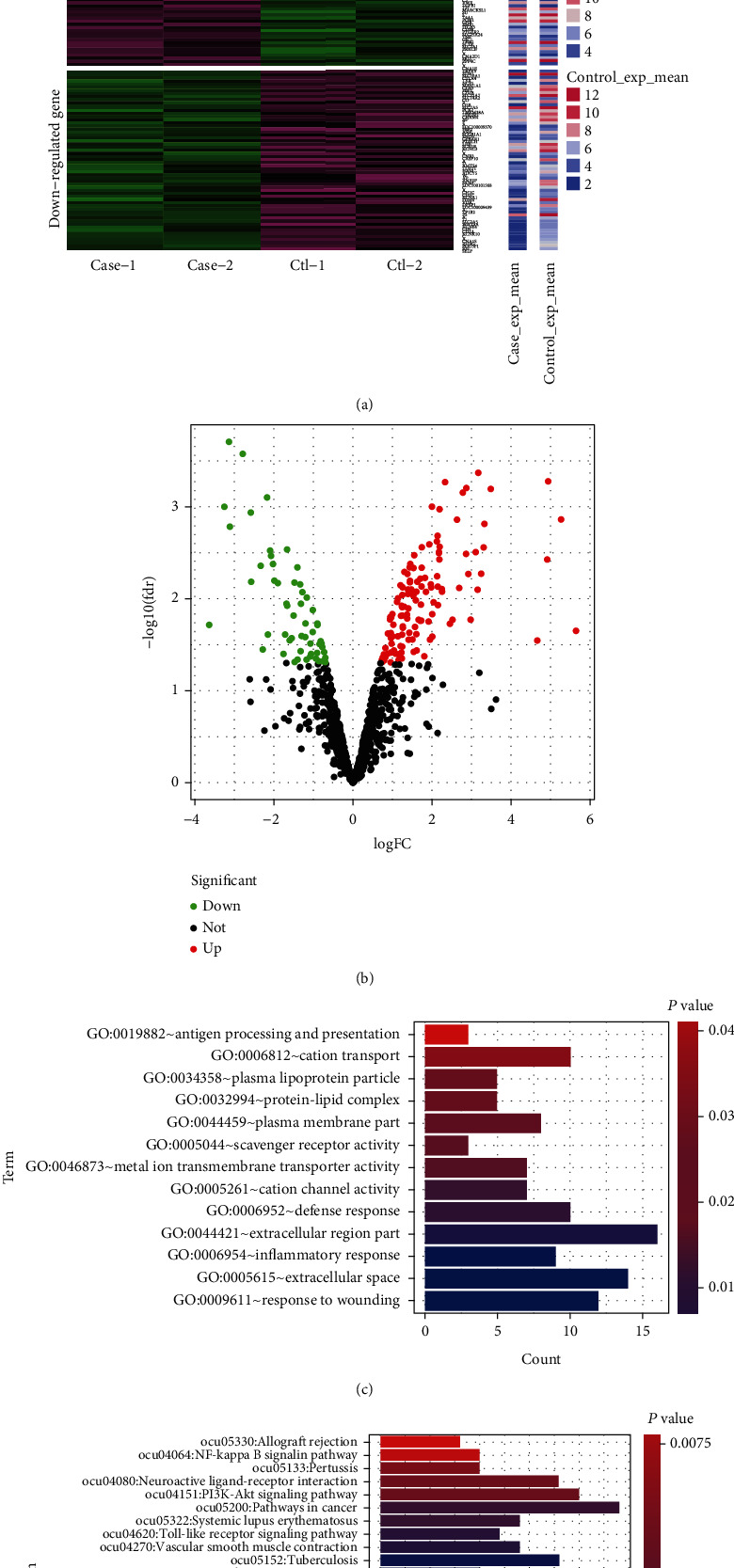
Screening results of differentially expressed genes in a rabbit pancreas tissue microarray. (a) Heat map of differentially expressed gene construction. (b) Volcano map of differentially expressed gene construction. (c) GO enrichment results of differential genes. (d) KEGG pathway enrichment results of differential genes.

According to the results of PCA between the AAC group and the control group, the signal peaks of the above metabolites were selected for statistical analysis, and the difference peaks were screened, with a *P* value < 0.05 serving as the threshold. The screening showed that there were significant differences in the changes in serum glucose (*δ* 3.90, *δ* 4.65), fat (*δ* 0.89, *δ* 2.24), glycoprotein (*δ* 2.04), and TMAO (*δ* 3.31). In this study, we primarily investigated the changes in serum TMAO (Figure 2(f)).

### 3.3. Differentially Expressed Gene Screening Results

To further study the effect of changes in serum metabolites on pancreatic transcription, we used chip sequencing data of pancreatic tissues in the normal control group and the AAC group to draw a volcano map under the conditions of *P* values < 0.05 and ∣log_2_FC | >0.58 (Figure 2(b)). A total of 182 differentially expressed genes were screened, including 123 upregulated genes and 59 downregulated genes. We devised a heat map based on differentially expressed genes (Figure 2(a)).

### 3.4. Differentially Expressed Gene Functional Enrichment Results

DAVID was used for GO enrichment and KEGG pathway analysis of differential genes. The results showed that GO enrichment of differentially expressed genes primarily involved such processes as cation transport and inflammatory response (Figure 2(c)), and the KEGG pathway primarily involved the NF-*κ*B pathway and tumor signaling pathway (Figure 2(d)). Among them, upregulated genes are involved in processes such as the NF-*κ*B pathway, cation transport, inflammatory response, and vascular smooth muscle contraction (Supplementary Figure S1a), and downregulated genes are involved in processes such as cation channel activity and bile secretion (Supplementary Figure S1b).

### 3.5. PPI Network and Network Node Topology Score Analysis

**Figure 3:**
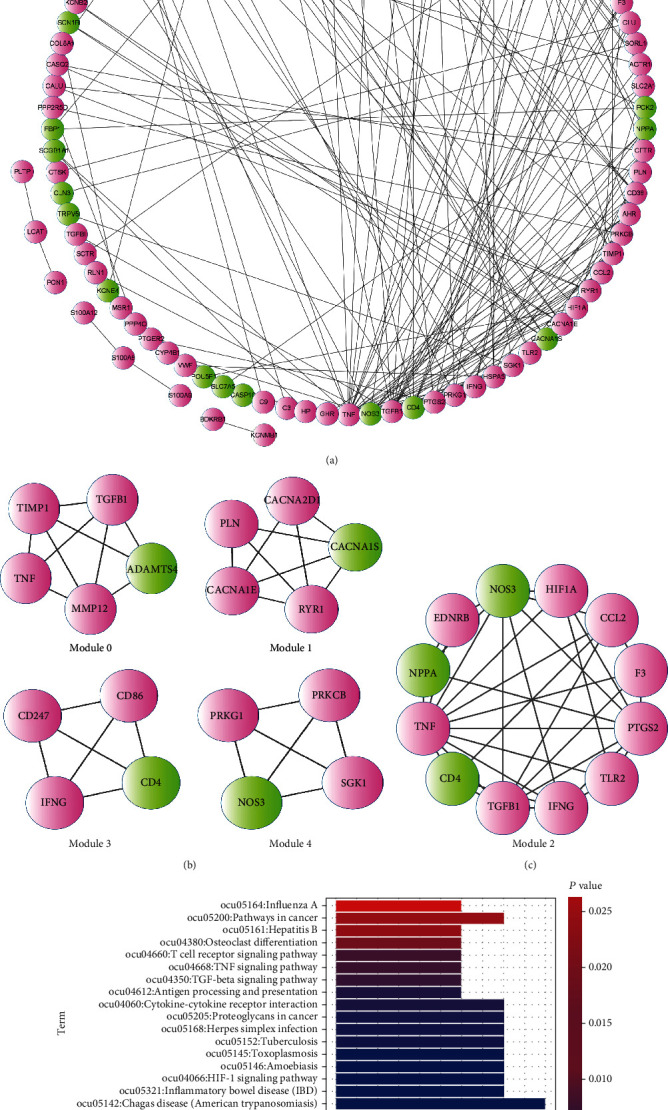
Protein interaction network analysis results of differential genes. (a) Protein interaction network analysis results of differential genes. (b, c) Five subnetwork modules found by CFinder software. (d) KEGG enrichment analysis results of module 2 network module node genes.

The STRING database was employed to construct a PPI network based on differentially expressed genes (Figure 3(a)). The network included 94 nodes and 185 interaction pairs. Subsequently, we calculated the degree centrality, betweenness centrality, subgraph centrality, and closeness centrality scores of each node.  Table 1 shows the top 15 nodes with the highest scores for the four centrality methods. Among these nodes, the top 3 nodes with the highest scores for each method were the TNF, NOS3, and TGFB1 genes.

### 3.6. Network Module Mining

We used CFinder software to mine 5 subnetwork modules (Figures 3(b) and 3(c)), of which only module 2 (Figure 3(c)) contained the three key differential genes TNF, NOS3, TGFB1, and the network module node gene KEGG functional enrichment showed that node genes were primarily involved in such pathways as the T cell receptor signaling pathway and the TNF signaling pathway (Figure 3(d)). In order to further explore the relationship between the results of this experiment and human AP, we analyzed the expression of three key genes TNF, NOS3, and TGFB1 in human pancreatitis tissues. Because there is no pancreatic tissue gene expression data for human acute pancreatitis in the GEO database, we analyzed the differential expression of genes in the mouse acute pancreatitis dataset GSE119844 and found that the differential expression trend of genes TNF, NOS3, and TGFB1 is consistent with that in this study (Supplementary Table S1), that is, in the inflammation samples, TNF and TGFB are upregulated, and NOS3 is downregulated. We also analyzed the differential expression of the three genes in the human chronic pancreatitis dataset GSE143754 and found that the differential expression trend of genes TNF and TGFB1 is consistent with that in this study, that is, TNF and TGFB1 were upregulated in inflammation samples (Supplementary Table S1).

## 4. Discussion

Acute acalculous cholecystitis (AAC) is an inflammation of the gallbladder not associated with the presence of gallstones. The main mechanisms of ACC are cholestasis [14] and gallbladder ischemia [15]. AAC has also been implicated as a cause of cholecystitis in previously healthy individuals. In this subgroup of patients, infectious causes comprise the primary etiology [16]. McChesney et al. proposed a progression from hypoperfusion and ischemia (from any cause, but commonly sepsis), to gallbladder inflammation, to resultant cholestasis and bacterial invasion, culminating in AAC [17]. Acute pancreatitis is a common life-threatening inflammatory disease of the pancreas, and biliary factors are the most common cause of pancreatitis. Through basic research and clinical trials, progress has been made in characterizing acute pancreatitis, which provides a basis for clinical treatment, but there are still many mechanisms of this disease that have not been fully elucidated. Many treatment options require clinical verification, and many potential therapeutic targets need to be tested in clinical trials [18–20]. At present, there are few studies on the genes and molecules related to the pathogenesis and progression of noncalculous biliary acute pancreatitis, either domestically or abroad, which limits the ability of healthcare providers to accurately diagnose and treat such cases of pancreatitis in the clinic. Therefore, this study analyzed the metabolomic and transcriptomic differences associated with AAC, examining the effects of serum metabolites on the pancreas during AAC, and provided a basis for the accurate diagnosis and treatment of noncalculous biliary acute pancreatitis.

This study showed that during AAC, the serum TMAO content was increased, and the differentially expressed genes in pancreatic tissue were enriched in such pathways as cation transport, inflammatory responses, the NF-*κ*B pathway, and the tumor signaling pathway. Among these genes, the expression of TNF and TGFB1 and the downregulation of NOS3 may be affected by serum metabolites and may play important roles in the pathophysiological process of acalculous biliary pancreatitis.

TMAO can be obtained directly from such foods as fish and shellfish [21], and choline, carnitine, and betaine can also be metabolized into trimethylamine (TMA) via intestinal microorganisms (primarily Clostridia, Proteus, Shigella, and Aerobacter) [22]. After that step, TMA is oxidized to produce TMAO by flavin-containing liver monooxygenases (FMOs), especially FMO3 [23]. Studies have found that TMAO levels exhibit a skewed distribution, indicating that the TMAO content in the human body is related to many factors, such as genetic background and eating habits, among individuals [24]. Meta-analysis showed that in cardiovascular diseases, diabetes, and other diseases, there was a nonlinear relationship between the increase in circulating TMAO concentration and the increase in C-reactive protein concentration (nonlinear = 0.015), and the circulating TMAO concentration was positively correlated with the risk of inflammation [25]. Zhang et al. [26] found that high levels of TMAO in the circulation downregulated the expression of the anti-inflammatory mediator RGS10 in peripheral tissues and the central nervous system, which may increase the activity of NF-*κ*B to induce inflammatory stimuli, thereby leading to increased production of inflammatory mediators and exacerbating peripheral tissue inflammation and inflammatory hyperalgesia. NF-*κ*B is responsible for the expression of many proinflammatory factors and chemokines, and the pathways from pathogen recognition to proinflammatory cytokine production have been demonstrated to be particularly dependent on NF-*κ*B [27]. Many other studies have shown that TMAO leads to a significant increase in the expression of the proinflammatory factors IL-1*β*, IL-6, and TNF-*α* by activating the p65 NF-*κ*B pathway which, in turn, causes inflammatory diseases [28–30], while the inhibitor nobiletin can significantly reduce TMAO-induced vascular inflammation by inhibiting the NF-*κ*B/MAPK signaling pathway [31]. The pathological increase in Ca^2+^ concentration in pancreatic acinar cells was the trigger point of acute pancreatitis [32]. Studies have shown that exposure to TMAO deteriorates myocardial cell mechanics and intracellular calcium processing capacity and reduces the efficiency of intracellular calcium removal [33]. High concentrations of TMAO can directly and significantly enhance the myocardial contractility of mouse and human heart tissues cultured *in vitro*. After acute treatment with TMAO, calcium fluorescence in myocardial cells was increased, and the increase in intracellular calcium ions was observed when TMAO influenced cell strength [34]. TMAO activated NLRP3 inflammasome and NF-*κ*B signaling to promote calcium/phosphorus-induced calcification of rat and human vascular smooth muscle cells in a dose-dependent manner and promoted vascular calcification [35]. In the pathogenesis of acute pancreatitis, calcium-dependent or calcium-independent signaling pathways are related to NF-*κ*B activation [36, 37]. The activation of NF-*κ*B can increase intracellular Ca^2+^ levels, thereby leading to Ca^2+^ overload [38] and inducing acute pancreatitis. In this study, compared with that in the normal control group, the TMAO in the rabbit serum of the AAC group was significantly increased. Therefore, we surmise that TMAO causes the expression of the proinflammatory factors IL-1*β*, IL-6, and TNF-*α* to increase significantly by activating the p65 NF-*κ*B pathway, which increases the level of Ca^2+^ in pancreatic acinar cells, inducing a sensitive state of pancreatitis.

The TNF gene encodes a multifunctional proinflammatory cytokine involved in the regulation of various biological processes, including cell proliferation, differentiation, apoptosis, lipid metabolism, and blood coagulation. It has been proven that TMAO can promote the production of the proinflammatory cytokines IL-1*β*, IL-6, and TNF-*α in vitro* [39, 40], and the activation of the NF-*κ*B pathway is necessary for TMAO to induce bone marrow mesenchymal stem cells to produce proinflammatory cytokines and release active oxygen [40]. TNF was determined to be closely related to acute pancreatitis. TNF-*α* can directly induce premature activation and necrosis of pancreatic acinar cell proteases, which depend on the activity of calcium ions and cathepsin-B [41]. Downregulating the expression of TNF-*α* can inhibit the release of inflammatory factors and plays a role in the treatment of acute pancreatitis [42, 43]. The protein encoded by the TGFB1 gene can regulate cell proliferation, differentiation, and growth and can regulate the expression and activation of other growth factors, including IFN-*γ* and TNF-*α*. TMAO is related to TGFB/SmAD signal transduction [44]. TMAO can increase the expression of TGFB receptor I (TGFBR1), activate the TGFBR1/Smad2 pathway, and aggravate myocardial fibrosis in mice [45]. The NOS3 gene (also known as eNOS) encodes nitric oxide synthase 3, which participates in the production of NO, while NO can serve as a biological mediator in the process of neurotransmission and antitumor activity. Studies have found that vascular endothelial cells have a protective effect on arterial contraction caused by TMAO. The arterial contraction induced by TMAO precursor TMA did not depend on perivascular adipose tissue or vascular endothelial cells and was mediated by nifedipine-sensitive calcium channels. This result suggested that vasoconstriction induced by metabolites may be physiologically important [46]. In cerulein-induced acute pancreatitis, NO derived from eNOS acts on nonacinar cells, possibly endothelial cells, to increase pancreatic microvascular blood flow, thereby exerting a protective effect [47]. Therefore, we surmise that the TNF and TGFB1 genes are upregulated by TMAO and that the TGFB gene participates in regulating the expression and activation of TNF, which increases the expression of the inflammatory factor TNF-*α* and induces a sensitive state of pancreatitis.

We obtained five subnetwork modules through protein interaction network module mining. The KEGG functional enrichment of the module 2 subnetwork module showed that node genes were mainly involved in such pathways as the T cell receptor signaling pathway, TNF signaling pathway, and TGFB signaling pathway. In the KEGG database, we found that the TNF gene directly participates in the T cell receptor signaling pathway, TNF signaling pathway, and TGFB signaling pathway, while these pathways are indirectly connected with the NF-*κ*B pathway, and the TNF gene directly participates in the NF-*κ*B pathway. Therefore, we surmised that elevated TMAO may increase the expression of inflammatory factors, such as TNF, through the NF-*κ*B pathway, and these inflammatory factors can participate in the NF-*κ*B signaling pathway, T cell receptor signaling pathway, TNF signaling pathway, TGFB signaling pathway, and other pathways, in turn. Therefore, a positive feedback loop was formed, which led to an increasing level of Ca^2+^ in pancreatic acinar cells, inducing a sensitive state of inflammation and increasing the risk of pancreatitis.

In summary, this study analyzed the serum metabolomics of rabbits with AAC and found that the serum TMAO content of the AAC group rabbits increased, and the change of TMAO content may facilitate the diagnosis of acalculous biliary pancreatitis. Furthermore, a total of 183 common differentially expressed genes were screened from the pancreatic tissue microarray data of the rabbit AAC model. The enrichment analysis results showed that differentially expressed genes were significantly enriched in such pathways as cation transport, the inflammatory response, the NF-*κ*B pathway, and vascular smooth muscle contraction. Based on the PPI network of differentially expressed genes, three key genes, TNF, NOS3, and TGFB1, were screened out. Through metabolomic analysis and functional analysis of differentially expressed genes, we realized that there was an association between serum metabolites and pancreatic gene expression during AAC, that is, TMAO activated NLRP3 inflammasome and NF-*κ*B signaling to promote calcium/phosphorus-induced calcification of rat and human vascular smooth muscle cells in a dose-dependent manner and promoted vascular calcification [35], and TMAO leads to a significant increase in the expression of the proinflammatory factors IL-1*β*, IL-6, and TNF-*α* by activating the p65 NF-*κ*B pathway [28–30], which, in turn, exacerbates AAC and induces a sensitive state of inflammation in pancreatic tissue. This association may be short-lived, or there may be a cascade. Although we cannot clearly explain the effect of amplification, these results may help to elucidate acalculous biliary pancreatitis from a new perspective, understand the characteristics of inflammation, and induce inflammation to occur and develop in a direction that is beneficial for humans.

## Figures and Tables

**Table 1:** The top 15 nodes with the highest scores for the 4 centrality methods.

Gene symbol	Subgraph	Gene symbol	Degree	Gene symbol	Betweenness	Gene symbol	Closeness
TNF	892.7949	TNF	26	TNF	2685.522	TNF	0.100108
NOS3	523.3804	NOS3	19	NOS3	2364.1072	NOS3	0.099893
TGFB1	510.1402	TGFB1	18	TGFB1	1224.675	TGFB1	0.098101
PTGS2	325.9173	PTGS2	11	PRKG1	998.8442	PTGS2	0.096774
IFNG	260.0609	CD4	11	HSPA5	857.3439	HSPA5	0.096774
CCL2	195.3008	PRKG1	10	PRKCB	670.7727	IFNG	0.096573
CD4	194.777	IFNG	9	RYR1	591.3694	CCL2	0.096573
TLR2	172.8715	HSPA5	9	MYLK	584.6416	SGK1	0.096373
HIF1A	165.2658	TLR2	8	CACNA1S	429.81485	TLR2	0.095975
SGK1	119.9916	HIF1A	8	CD4	418.01648	HIF1A	0.095778
F3	101.6659	SGK1	8	SGK1	409.25357	PRKG1	0.095679
NPPA	100.6601	CACNA1S	8	CLU	368.7241	MYLK	0.095483
PRKG1	99.27879	CACNA1E	8	CD59	334	NPPA	0.094898
HSPA5	96.47914	CCL2	7	ADCY5	332	EDNRB	0.094608
TIMP1	79.86854	RYR1	7	MYH11	332	PRKCB	0.094608

## Data Availability

The data used to support the findings of this study are available from the corresponding author upon request.

## Supplementary Materials

Supplementary Materials Figure S1 and Supplementary Table S1-3 are presented in Supplementary Material.
