# Supplementary material for: Effects of Serum Metabolites on the Pancreatic Transcriptome in Acute Acalculous Cholecystitis
Source: Gastroenterol Res Pract. 2021 Dec 8;2021:2368571. doi: 10.1155/2021/2368571 (PMC8674085; doi:10.1155/2021/2368571)
Supplement: Supplementary Materials — Figure S1 and Supplementary Table S1-3 are presented in Supplementary Material. [file 2368571.f1.docx]

| Gene | Acute pancreatitis (Mouse,GSE119844) | | Chronic pancreatitis (Human,GSE143754) | |
| --- | --- | --- | --- | --- |
|  | logFC | p-value | logFC | p-value |
| TNF | 2.06109 | 0.01305 | 0.42378 | 0.00024 |
| TGFB1 | 1.56797 | 0.00037 | 0.28938 | 0.08630 |
| NOS3 | -0.58453 | 0.07500 | 0.28874 | 0.00139 |

Table S1 Expression of genes TNF,TGFB1,NOS3 in acute pancreatitis and chronic pancreatitis.

Table S2 The logFC and p values of down-regulated genes among differentially expressed genes in rabbit pancreatic tissue.

| **genesymbol** | **logFC** | **P.Value** | **genesymbol** | **logFC** | **P.Value** |
| --- | --- | --- | --- | --- | --- |
| SELP | -3.63821 | 0.019075 | KCNC3 | -1.33657 | 0.006927 |
| RS1 | -3.25434 | 0.000985 | SCN1B | -1.32519 | 0.036967 |
| POU5F1 | -3.13764 | 0.000193 | LHB | -1.31627 | 0.011258 |
| RHBG | -3.11389 | 0.001622 | PTHLH | -1.30081 | 0.025255 |
| CACNA1S | -2.78925 | 0.000262 | GPBAR1 | -1.27947 | 0.008424 |
| KCNK10 | -2.58517 | 0.001139 | ENO3 | -1.20743 | 0.025958 |
| CD1A | -2.57603 | 0.006469 | SCGB1A1 | -1.20132 | 0.018446 |
| CD4 | -2.33468 | 0.004337 | ASIP | -1.17395 | 0.045734 |
| KCNE4 | -2.28244 | 0.035377 | FBP2 | -1.16796 | 0.009651 |
| WNT5A | -2.17286 | 0.000782 | LOC100009370 | -1.08211 | 0.042328 |
| SLC2A5 | -2.15311 | 0.024322 | NPPA | -1.07647 | 0.030544 |
| PSAT1 | -2.09575 | 0.00296 | CHRM4 | -1.05876 | 0.039636 |
| ATP1B2 | -2.07231 | 0.00338 | UBE2R2 | -1.01843 | 0.022885 |
| LOC100009439 | -2.02299 | 0.00415 | TMEM38A | -1.0165 | 0.013199 |
| DPEP1 | -1.98809 | 0.006295 | PCK2 | -0.93781 | 0.044553 |
| VDR | -1.90098 | 0.006689 | SLC7A5 | -0.90752 | 0.038675 |
| ITIH4 | -1.75757 | 0.039582 | CORO1B | -0.89997 | 0.018531 |
| KCNA1 | -1.73922 | 0.022668 | SLC14A2 | -0.89783 | 0.0192 |
| CD1C | -1.71694 | 0.024324 | SLC22A7 | -0.86704 | 0.047559 |
| CACH2C | -1.68602 | 0.04976 | CD1B | -0.84438 | 0.030964 |
| LOC100101568 | -1.68231 | 0.011227 | FBP1 | -0.82177 | 0.030472 |
| NOS3 | -1.66746 | 0.002887 | CLN3 | -0.81922 | 0.030987 |
| ANPEP | -1.66513 | 0.011808 | MAN1A1 | -0.81718 | 0.02888 |
| WAP | -1.60142 | 0.028155 | NCF1 | -0.79503 | 0.030699 |
| ADCY5 | -1.55098 | 0.02668 | TYR | -0.77607 | 0.033441 |
| HAS3 | -1.50257 | 0.01512 | CTLA4 | -0.76217 | 0.047768 |
| TUFT1 | -1.48084 | 0.00662 | SLC10A1 | -0.72909 | 0.037968 |
| ADAMTS4 | -1.47543 | 0.048496 | EDN1 | -0.70083 | 0.04891 |
| CASP10 | -1.40583 | 0.004522 | TRPV5 | -0.70008 | 0.043768 |
| CACNB3 | -1.4009 | 0.045643 |  |  |  |

Table S3 The logFC and p values of up-regulated genes among differentially expressed genes in rabbit pancreatic tissue.

| **genesymbol** | **logFC** | **P.Value** | **genesymbol** | **logFC** | **P.Value** |
| --- | --- | --- | --- | --- | --- |
| CACNA1E | 0.729695 | 0.045994 | MYOZ1 | 1.55024 | 0.003335 |
| PPP4C | 0.739038 | 0.048727 | TNFRSF4 | 1.569713 | 0.020101 |
| SELL | 0.751821 | 0.043463 | ARG1 | 1.590182 | 0.010327 |
| CACNA2D1 | 0.78215 | 0.040168 | IFNG | 1.604476 | 0.024094 |
| PRKCB | 0.786205 | 0.046756 | CD3E | 1.604897 | 0.008322 |
| SLC2A1 | 0.820461 | 0.043031 | SORL1 | 1.612404 | 0.036251 |
| SCTR | 0.820485 | 0.034204 | ACTA2 | 1.627354 | 0.006477 |
| TPM1 | 0.880715 | 0.039368 | SFRP2 | 1.661 | 0.01679 |
| GJC1 | 0.883451 | 0.023807 | NCF4 | 1.703355 | 0.01725 |
| TNF | 0.936212 | 0.016708 | TLR2 | 1.705163 | 0.005992 |
| SLC25A24 | 0.944001 | 0.015732 | LOC100009332 | 1.719535 | 0.011487 |
| PTGER2 | 0.953531 | 0.048951 | PPP2R5D | 1.727974 | 0.017156 |
| CD38 | 0.953871 | 0.025428 | SGK1 | 1.734923 | 0.007285 |
| ITGB3 | 0.956563 | 0.023516 | TPM2 | 1.738686 | 0.004552 |
| EMP1 | 0.957002 | 0.038311 | APOBEC1 | 1.742085 | 0.002729 |
| GHR | 0.959429 | 0.026599 | ART4 | 1.805091 | 0.041987 |
| PON1 | 0.968674 | 0.015888 | PLN | 1.822746 | 0.005872 |
| SULT4A1 | 0.976863 | 0.030681 | MSR1 | 1.846702 | 0.008331 |
| MARCKSL1 | 0.996887 | 0.014621 | KCNMB1 | 1.913378 | 0.017554 |
| TH | 1.001705 | 0.025532 | VCAM1 | 1.93055 | 0.002537 |
| TGFBI | 1.021905 | 0.019065 | HSPA5 | 1.94859 | 0.027658 |
| VWF | 1.037327 | 0.036351 | PRKG1 | 1.970176 | 0.006911 |
| IMPACT | 1.077886 | 0.03943 | DKK1 | 1.980534 | 0.007447 |
| MYLK | 1.115431 | 0.010751 | CASQ2 | 1.999039 | 0.000986 |
| LOC100008640 | 1.136175 | 0.025463 | ACTA1 | 2.005616 | 0.014577 |
| RYR1 | 1.137971 | 0.009835 | KITLG | 2.007069 | 0.025626 |
| SLC4A9 | 1.14613 | 0.040014 | PLTP | 2.039959 | 0.010826 |
| TFPI | 1.151371 | 0.046432 | AOAH | 2.122466 | 0.002352 |
| CDKL2 | 1.202462 | 0.007098 | MYH11 | 2.129668 | 0.005632 |
| S100A11 | 1.204968 | 0.032544 | BGN | 2.141337 | 0.002043 |
| KCNB2 | 1.215423 | 0.015239 | S100A8 | 2.146586 | 0.00731 |
| PTGS2 | 1.226122 | 0.038289 | EDNRB | 2.149649 | 0.01162 |
| ITGB8 | 1.233566 | 0.011851 | C1R | 2.174375 | 0.003147 |
| ADH1A | 1.237293 | 0.044319 | CLU | 2.175909 | 0.00307 |
| CALD1 | 1.243064 | 0.012255 | S100A9 | 2.186018 | 0.003693 |
| MMP12 | 1.243824 | 0.02445 | LOC100125995 | 2.191007 | 0.00105 |
| AHR | 1.245657 | 0.033014 | RLN1 | 2.192502 | 0.00269 |
| PTGER4 | 1.248402 | 0.01513 | SGCA | 2.24952 | 0.007769 |
| ITIH3 | 1.249387 | 0.009046 | TIMP1 | 2.253741 | 0.008287 |
| LPXN | 1.255557 | 0.007468 | CD86 | 2.330105 | 0.000532 |
| ABCC9 | 1.261378 | 0.019839 | CD14 | 2.452999 | 0.01855 |
| C9 | 1.262089 | 0.020574 | BDKRB1 | 2.518709 | 0.016774 |
| TGFB1 | 1.297546 | 0.012141 | TNFAIP6 | 2.631962 | 0.001363 |
| CD247 | 1.302059 | 0.005077 | FPR1 | 2.687568 | 0.007553 |
| CTSK | 1.366943 | 0.014781 | ORM1 | 2.779858 | 0.00069 |
| PTH1R | 1.371536 | 0.016269 | PTGS1 | 2.861799 | 0.003211 |
| CFTR | 1.377559 | 0.005328 | CTSE | 2.86849 | 0.000616 |
| AGTR1 | 1.391839 | 0.008795 | SPP1 | 2.918287 | 0.005315 |
| F3 | 1.393295 | 0.007869 | DMBT1 | 2.976781 | 0.016749 |
| PLA2R1 | 1.401685 | 0.012653 | LOC100008716 | 3.105927 | 0.003072 |
| CALU | 1.407059 | 0.027718 | CYP4B1 | 3.154001 | 0.007883 |
| CCL2 | 1.424912 | 0.023275 | ACP5 | 3.170285 | 0.00042 |
| CCL4 | 1.433683 | 0.004527 | ANXA8 | 3.24395 | 0.005275 |
| SLC6A8 | 1.44162 | 0.006576 | COL8A1 | 3.306025 | 0.002728 |
| HIF1A | 1.442529 | 0.006326 | S100A12 | 3.326298 | 0.001512 |
| LCAT | 1.450185 | 0.004154 | IGKC1 | 3.487004 | 0.000629 |
| CD5 | 1.475285 | 0.007862 | LOC100009259 | 4.663112 | 0.02824 |
| C3 | 1.47879 | 0.008089 | LOC100009168 | 4.914505 | 0.003694 |
| RBP4 | 1.505446 | 0.027548 | HP | 4.938486 | 0.000519 |
| CD59 | 1.527541 | 0.009184 | LOC100009097 | 5.266196 | 0.001352 |
| CTSD | 1.532673 | 0.00459 | SAA3 | 5.644115 | 0.022136 |
| PPARD | 1.537192 | 0.037595 |  |  |  |


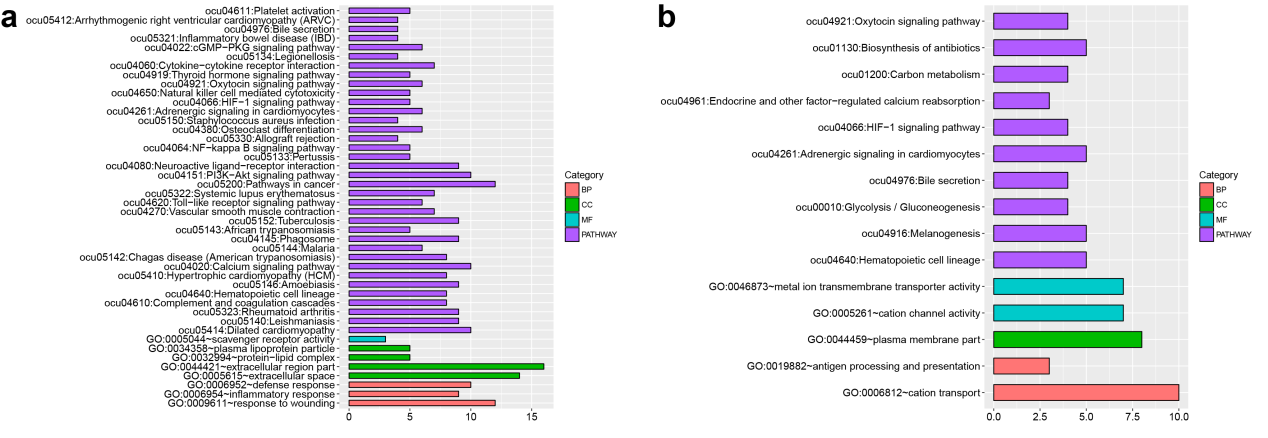


Figure S1: **Screening results of differentially expressed genes in a rabbit pancreas tissue microarray (a): GO enrichment results and KEGG pathway enrichment results of** up-regulated **differential expression genes; (b): GO enrichment results and KEGG pathway enrichment results of** down-regulated **differential expression genes.**
